# Capability accumulation patterns across economic, innovation, and knowledge-production activities

**DOI:** 10.1038/s41598-023-29979-x

**Published:** 2023-08-10

**Authors:** Aurelio Patelli, Lorenzo Napolitano, Giulio Cimini, Emanuele Pugliese, Andrea Gabrielli

**Affiliations:** 1 Enrico Fermi Research Center, Rome, Italy; 2 European Commission, Joint Research Centre (JRC-Seville), Seville, Spain; 3 Physics Department and INFN, University of Rome Tor Vergata, Rome, Italy; 4 Department of Civil, Computer Science and Aeronautical Technologies Engineering, Università degli Studi “Roma Tre”, Rome, Italy

**Keywords:** Complex networks, Social evolution, Scientific data

## Abstract

The evolution of economic and innovation systems at the national scale is shaped by a complex dynamics related to the multi-layer network connecting countries to the activities in which they are proficient. Each layer represents a different domain, related to the production of knowledge and goods: scientific research, technology innovation, industrial production and trade. Nestedness, a footprint of a complex dynamics, emerges as a persistent feature across these multiple kinds of activities (i.e. network layers). We observe that, in the layers of innovation and trade, the competitiveness of countries correlates unambiguously with their diversification, while the science layer shows some peculiar features. The evolution of the scientific domain leads to an increasingly modular structure, in which the most developed countries become relatively less active in the less advanced scientific fields, where emerging countries acquire prominence. This observation is in line with a capability-based view of the evolution of economic systems, but with a slight twist. Indeed, while the accumulation of specific know-how and skills is a fundamental step towards development, resource constraints force countries to acquire competitiveness in the more complex research fields at the expense of more basic, albeit less visible (or more crowded) ones. This tendency towards a relatively specialized basket of capabilities leads to a trade-off between the need to diversify in order to evolve and the need to allocate resources efficiently. Collaborative patterns among developed countries reduce the necessity to be competitive in the less sophisticated research fields, freeing resources for the more complex ones.

## Introduction

National economic systems can be represented as a network of interrelated layers, each referring to a characteristic domain of activities (e.g. trade, innovation, scientific research), a subset of which are measurable. Indeed, the view of economic outcomes as the result of the complex interactions between interconnected systems is a long-standing idea with deep roots e.g. in evolutionary economics. One notable example is the notion of systems of innovation^1^, defined as the ensemble of private and public institutions operating within a territory that concur, through their activities and mutual relations, in the discovery and spread of new technologies. The concept of innovation systems has been adapted successfully over time into a framework to describe and analyze National^2,3^ as well as Regional systems^4^. Universities, firms, and the public sector are among the relevant actors in this framework because they are responsible for the interactions that shape innovation and knowledge transmission. The activities of this micro-actors are aggregated at the level of cities and regions^5^, and finally in countries—the central unit of our analysis.

A similar perspective is shared by the triple helix model^6^ and its later refinements, which identify economic agents and their co-evolution as drivers of knowledge production and innovation in knowledge-based societies, while reducing the emphasis on the importance of borders and local specificities. As a result, understanding how the different layers evolve over time has become increasingly relevant in the economic literature and the variety of empirical tools proposed to address it has grown accordingly.

Economic Complexity^7,8^ provides a framework to study innovation systems analysing how countries expand their skills in domains of activities (e.g. scientific research) that can be encoded as bipartite networks connecting each country to measurable outputs of said activities (e.g. publications by scientific field) and how these evolving skills are related.

For instance, the interaction between science, technology and production is rich and displays a high degree of interconnection. Indeed, the strongest statistically significant signal of interaction between these layers of activities suggests that technological breakthroughs drive the development of new products and science^9^, although the sub-leading interactions are also significant.

Each layer displays specific features, some of which are connected to the main drivers of capability accumulation within countries, which result in different countries acquiring a competitive edge in different sets of outputs and, consequently, leads to different diversification patterns within layers. Nestedness^10^ is a measure of the global structure of bipartite networks that allows to capture differences in their diversification patterns and has been shown to shed light on the dynamics underlying their evolution in applications to economic data^11^. For instance, in a nested system, more specialized countries have in their portfolio of outputs a subset of the outputs of more diversified countries. Economic Fitness and Complexity is particularly effective in bringing out nested patterns in bipartite network data^12^. Therefore,the aim of this paper is to apply techniques grounded in Economic Fitness and Complexity to measure the structural evolution of each layer, as captured by their degree of nestedness, and compare the layers with one another.

It is noteworthy that the scientific literature about biology and ecological systems provides a further foundation for our analysis because mutualistic systems create patterns comparable to the ones found in economic systems.

Therefore, exploiting the analogy by applying the methods borrowed from the ecological literature could help shed light on interesting properties of human systems, possibly related to their stability and evolution.

The manuscript starts by introducing the material and methods employed in this paper, describing the data and the metrics on which we base the empirical analysis. The main results are presented in detail in the following eponymous section, while the final section discusses our findings and their implications.

## Materials and methods

This section describes the databases considered in the analysis and the tools and methods implemented.

### Databases

The analysis of the scientific layer is based on data that aggregates the scientific output of countries into scientific fields. The source of this data is the Open Academic Graph v2 (OAG)^13–15^ which is a snapshot of the Microsoft database taken in the late November 2018. OAG lists a large number of academic papers, reporting for each one information such as its citation counts and the institute or university to which the authors are affiliated. For instance, the dataset we consider is composed by 55513517 scientific documents with 809963530 citations. The database covers most of the journals, conference proceeding, books and manuscripts published from the early 1800s up to the moment when the snapshot was taken. We consider publications starting from 1960 because in earlier years only a small number of publications is available, which mostly concentrate in a small set of developed countries. In terms of geographical coverage, OAG accounts for most of the economies in the world and it is one of the most complete and detailed datasets in terms of the geographical coverage it allows. OAG presents a small bias towards developed and English-speaking countries^16^, although this is a common characteristic of many other databases, e.g. Scopus^17,18^.

The RegPat^19^ database, built by the OECD and updated yearly, accounts for patented inventions being created by applicants and inventors located within their territorial classification. The database does not cover developed countries uniformly and, since it focuses on patent applications submitted to the European Patent Office (EPO), it has a bias toward Europe. Nevertheless, the database makes up for this shortcoming thanks to an accurate geocoding of the patent documents it contains. Overall, it contains data about 200 countries and 649 4-digits technological codes of the CPC classification, aggregating 3484918 patents families. It covers the period from 1978, when the EPO was first established, to the year of publication. For the present analysis, we use patent data to build the invention layer of the innovation system, which we employ as a proxy for technological innovation taking place within countries. To an extent, this entails a simplification of a more complex phenomenon, which takes place in many contexts, many of which are not as easily codified. This issue is well-known in the innovation literature, and many contributions have tried to address this shortcoming by exploring alternatives to patent data. However, for our purposes, it is necessary to have a granular view of the scope of innovation outputs as well as a standard classification to compare them across countries. To our best knowledge, data about filed patents and the associated technology codes are the only available data source satisfying all these requirements while providing international coverage. For this reason, though we are aware of the limitations imposed by data availability, throughout the manuscript we proxy technological innovation with patented inventions.

The COMTRADE^20^ database collected by the UN, which reports the trade flows of physical goods between countries, forms the basis for the economic layer employed in our analysis. The database, as homogenized by^21^, covers 169 national economies and reports 1218 4-digits product codes of the HS-1992 classification. The size of the database is not directly available but typically there are from $10^6$ to $10^7$ trade report flows per year.

### Measures of capability accumulation patterns

The technological impact of a country can be measured with the number of patents filed by field of technology and economic impact can be measured with export flows by product category. Scientific impact is instead usually based on citation counts because citations are widely recognized as a proxy of the quality of the research performed by authors, institutions and, consequently, countries. However, due to the rich-get-richer mechanism, citation counts display very skewed distributions with fat tails and weak convergence to stationary measures^22–24^. To partly correct skewness one can employ *log-citation counts*^16,25,26^, defined as

1 $$\begin{aligned} {[}lcit]_{i\alpha } = \log (1+[cit]_{i\alpha }). \end{aligned}$$

where the label *i* refers to geographical areas and the label $\alpha$ refers to the scientific field. In the present manuscript we consider both citation counts and log-citations counts. As shown below, the latter choice yield results that are both more stable (with lower fluctuations) and easier to interpret.

A meaningful comparison between countries requires identifying the outputs within a domain of activity, in which each country has a well-established skill set. To this aim, it is useful to filter out observations that could be the result of contingency or fluctuations. For instance, a one-off scientific publication in the field of, say, quantum computing in a country would register in the data. However, one could hardly argue that it would be a strong signal of the country having strong capabilities in the field. This kind of filtering can be achieved in several ways. A popular option in the literature is the Revealed Comparative Advantage (RCA) indicator^27^, commonly used as a measure of relative specialization. As shown in Eq. (2), RCA is computed as the weight of an activity in country baskets of activities relative to the global weight of the same activity.

2 $$\begin{aligned} \text {RCA}_{i\alpha } = \frac{W_{i\alpha }}{\sum _{\beta } W_{i\beta }} \Big / \frac{\sum _j W_{j\alpha }}{\sum _{j\beta } W_{j\beta }} \end{aligned}$$

where $W_{i\alpha }$ indicates the extensive measure over which the RCA is computed, i.e. patent counts, export flows or log-citations, depending on the layer.

RCA takes values on a continuum and can hence encode a great deal of information. However, for our purposes a more *coarse grained* measure is more adequate. For this reason, we transform RCA into binary values flagging the activities in which each country is more proficient than a given threshold. Following standard practice, we apply binary filtering by keeping only RCA values above ${\text {RCA}}^{*}=1$, thus constructing binary bipartite networks whose adjacency matrix has elements $M_{i\alpha }=\Theta (\text {RCA}_{i\alpha }-1)$, where $\Theta (\cdot )$ is the Heaviside step function $\Theta (x)=1$ if $x\ge 0$ and 0 otherwise. It is worth mentioning that the results obtained via the Economic Complexity methodology are robust to other binarization strategies that do not involve RCA. In fact, any approach yielding a binary adjacency matrix, while preserving its structure, is a reasonable candidate. In other words, the constraint we face at this stage is to preserve the information about *what* countries significantly do, in contrast to *how much* (this rules out well-established competitiveness indices such as the Global Competitiveness Index^28^ that yield an aggregate score at the country level.). However, a noticeable advantage of employing RCA is that it allows to apply a consistent methodology to all layers, which allows inter-layer comparability without the need of *ad hoc* assumptions e.g. about where to set the binarization threshold.

### Nestedness

Nestedness is a property of systems consisting of actors with heterogeneous features that measures the extent to which shared features belong to both feature-rich and feature-poor actors. In the manuscript we estimate the nestedness of each network through the computation of two metrics widely used in literature, the *Temperature of Nestedness* (Throughout the study, we also refer to this measure as Nestedness Temperature or Temperature)^29,30^ and the *Nestedness metric based on Overlap and Decreasing Fill* (NODF)^31^. NODF estimates the nestedness evaluating the overlap of each row and column with respect to all the others rows and columns. Defining ${\textbf{M}}$ the bi-adjacency matrix of the network considered, NODF is computed as

3 $$\begin{aligned} NODF({\textbf{M}}) = \frac{1}{{\mathbb {N}}}\left[ \sum _{ij}\Theta (k_i-k_j)\frac{C_{ij}}{k_j} + \sum _{\alpha \beta }\Theta (k_\alpha -k_\beta )\frac{C_{\alpha \beta }}{k_\beta } \right] \end{aligned}$$

where $k_i=\sum _\alpha M_{i\alpha }$ and $k_\alpha =\sum _i M_{i\alpha }$ are respectively the number of ones of rows *i* and column $\alpha$ (the degree of the corresponding network nodes), $C_{ij}=\sum _\alpha M_{i\alpha }M_{j\alpha }$ is the number of co-occurrences between of rows *i* and *j*, and $C_{\alpha \beta }=\sum _i M_{i\alpha }M_{i\beta }$ is the number of co-occurring element between columns $\alpha$ and $\beta$. ${\mathbb {N}}$ is a suitable normalization while $\Theta (x)$ is the step function. The two terms inside the square brackets are proportional to the row NODF and the column NODF respectively.

On the contrary, the Temperature of Nestedness is computed through a rather convoluted formula evaluating the unexpectedness of 0/1 at the distance above/below the isocline coinciding with the line of perfect nestedness, which is determined by the density of the adjacency matrix. We use the code made available by the Nestedness for Dummies (NeD) project (The code for this EU-funded project is available at http://ecosoft.alwaysdata.net/) to compute the Nestedness Temperature.

Notice that nestedness, as well as modularity in the next section, is an aggregate property of the system, not a property of specific countries. Indeed in the present analysis we are not looking at the evolution of each different country and their heterogeneous trajectories^32–34^, but only at the emerging shared properties of the dynamical process. In general, it is of crucial importance for analyses based on the Economic Fitness and Complexity framework that there is enough heterogeneity among the actors comprising the systems to obtain meaningful results^35^.

### Modularity

Modularity measures the quality of a partition of a network, i.e. a given community structure. According to the modularity metric, the best community structure maximizes

4 $$\begin{aligned} Q=\max _{\xi }\left\{ \frac{1}{N}\sum _{ij}\left( A_{ij}-\frac{k_ik_j}{2N}\right) \delta (\xi _i,\xi _j) \right\} \end{aligned}$$

where $\xi _i$ is the label of the partition to which node *i* belongs and $A_{ij}$ is the adjacency matrix of the network with *N* nodes. In other words, modularity measures the number of network links within the communities, with respect to a random benchmark. In this work we compute the modularity of the monopartite projections of the bipartite networks ${\textbf{M}}$ corresponding to the layers connecting countries to their activities. We focus mainly on the evolution of the modularity of each layer, where the elements of the adjacency matrix ${\textbf{A}}$ of Eq. (4) are obtained as $A_{ij}=\Theta [C_{ij}]$, i.e. the binarized co-occurrences of the various activities in pairs of countries. Moreover, we check that the modularity of the monopartite representation of each bipartite network, often considered in literature about the block nestedness, does not create meaningful partitions in terms of specialized blocks^36^. The value of the modularity of the best partitioning measures the inter-dependence between the modules found, thus providing an estimate of the strength and stability of the proposed communities.

## Results

**Figure 1:**
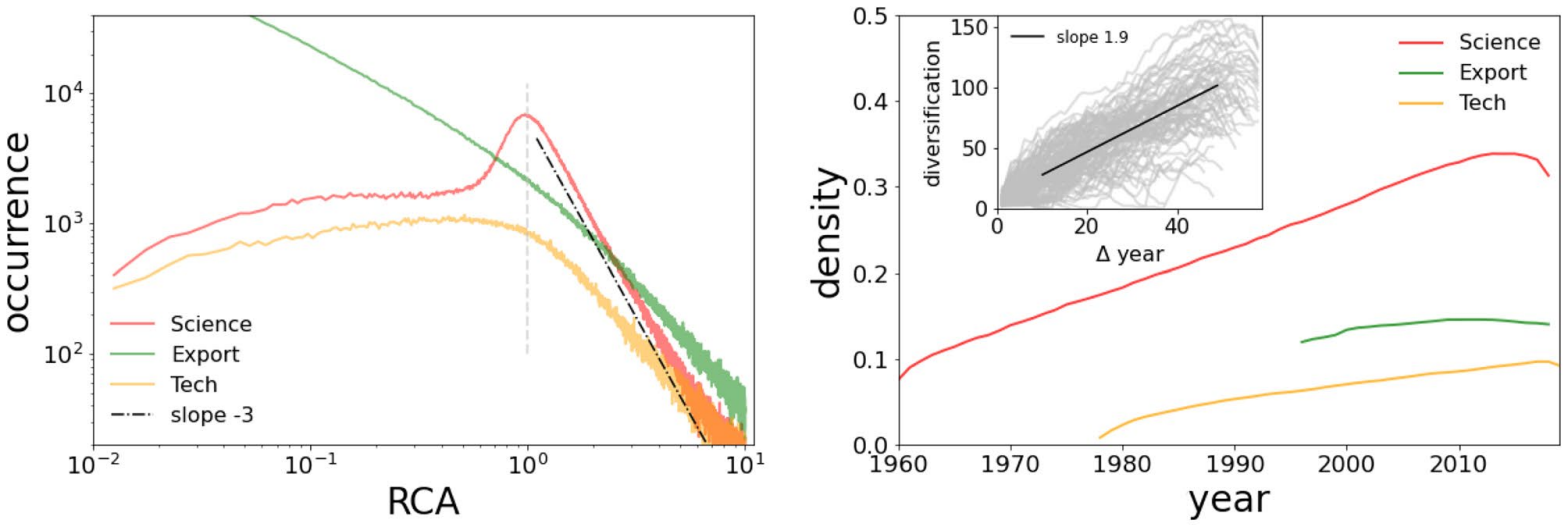
The left panel shows the occurrence of RCA for the different layers in log scale. The dashed line indicates the position of the threshold value of ${\text {RCA}}^{*}=1$, while the full black line is an eye-guidance for a slope of $-3$. The right panel shows the density of elements having RCA larger than 1 in the different layers considered: the Scientific (red line), the Technological (orange line) and the International Trade Export (green line). The inset indicates the growth of the scientific diversification of the different countries with respect to the initial time of production.

As explained in the “Materials and methods” section, the competitiveness of each country in a given production-related or innovation-related activity can be estimated by evaluating the RCA of the country in that activity. The RCA values associated with the economic layer are usually based on international trade data because the country’s exports of a good are considered an indicator of the competitiveness of the industry producing that good. Globally, the vector of RCA values of a country in the economic layer contains information about the capabilities that the country has in the associated domain of activity. The same is true for the technological layer, where RCA values can be computed on the number of patents filed by technological field, and for the scientific layer where RCA can be computed from log-citation counts by scientific field. The former layer is informative of national capabilities in the domain of technological innovation, while the latter is informative of national capabilities in the scientific domain. Therefore, the spectra of RCA values differ across layers (see Fig. 1, right panel). For example, the profile of the RCA distribution of the scientific data peaks around 1, with a lower occurrence of low RCA values and a stronger power-law decay corresponding to large RCA values. Conversely, the economic data does not display a peak in the analyzed spectrum.

For all layers, the power-law decay is a sign that the system is highly heterogeneous since it cannot be easily replicated by the RCA distribution obtained by considering random matrices. Yet, the slope of the decay depends on the particular properties associated with the relative competitiveness of the countries^37^. Interestingly, the slope of the RCA distribution of the Technological and Scientific layers have a cross-over around 1 (the global average) while the distribution of the RCA of the international trade exports does not present a clear cross-over.

The binary representation of the RCA intrinsically defines the bipartite networks describing the competitiveness of the countries in the activities characterizing each layer. A basic quantity of interest in this representation is the network density, defined as the fraction of observed links with respect to the maximum number, i.e. the number of links one would observe in the fully connected graph. The time series of the density of the Scientific and Technological layers indicates that the countries increase their diversification as time increases, as shown in the right panel of Fig. 1. This growth marks a second difference of Science with respect to the Production layer, which features a much steadier evolution of diversification. Indeed, the density of the scientific environment grows almost linearly and it is probably triggered by the exponential growth of the scientific corpus with a doubling period of approximately a decade^38^. Such exponential growth is not found only at the global scale but also at the country level for most of the developed and developing countries (as shown in the inset of the right panel of Fig. 1). A similar pattern can be observed in the production of patents, while it is not reproduced in the available Export data in the last decades, but it was detected during the economic boom around the sixties^39^.

**Figure 2:**
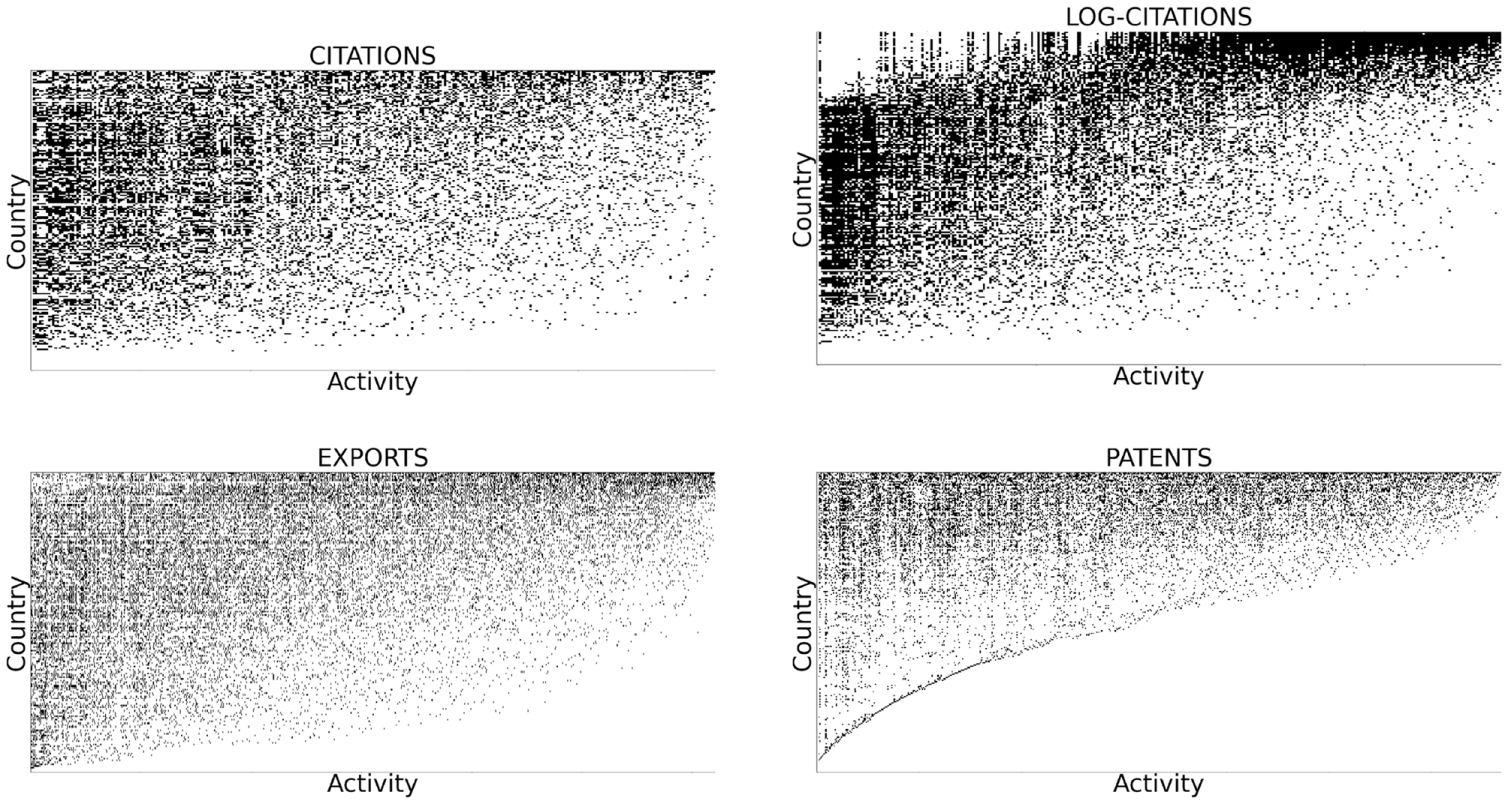
The binary matrices in 2000, obtained by thresholding the RCA at 1, for the different layers: the scientific citation (top left), the log-citation (top right), the export flow (bottom left) and the patent production (bottom right).

The most important and characteristic pattern emerging from the binary representation is the presence of the triangular shape of the matrix ${\textbf{M}}$, visible when the rows and columns are properly ordered^7,8^. All the layers display the hierarchical structure shown in Fig. 2, where top rows, corresponding to the countries with the most advanced set of capabilities, show high diversification; furthermore, some activities are highly ubiquitous (leftmost columns) while others are performed only by the top countries (rightmost columns). This characteristic *triangularity*, which becomes apparent when the matrices are properly ordered, signals a high *nestedness*, though, unfortunately, a precise mathematical definition of nestedness is still lacking^40^.

Among the innovation-related layers, science appears to be the least nested (i.e. the one with least triangular adjacency matrix), while the technological layer displays a relatively sharp boundary along the diagonal, highlighting its nested structure nicely within the associated adjacency matrix. This feature points to a structure of the scientific layer that presents more intrinsic heterogeneity, compared to the other ones. Such heterogeneity was considered as the cause of the lower quality of the scientific layer in the context of the Economic Fitness and Complexity^18,41^, which was solved by the introduction of the more stable log-citations^16^ metrics. Interestingly, the network based on the log-citations metrics presents a hole in the top left corner, which is not observed in the other cases, suggesting that the top countries are not the most competitive in scientific fields with high ubiquity. Note that the absence of the top left links does not mean that the top countries are not producing science in the less complex scientific fields; rather, it suggests that the number of citations they receive in those fields is below the fair share, given the global average (Indeed, looking at the value of the RCA in the hole region, the values are constantly close to one, but consistently below.).

The lack of a precise definition of nestedness induces the derivation of different, albeit slightly counter-intuitive, metrics. Indeed, depending on the feature considered as the representative characteristic of nestedness, various metrics can be defined to make the concept operational. In this work we consider the Temperature of Nestedness^29^, and the NODF^31^ because both are connected to different, yet related, features characterizing the dynamics of the innovation layers. The main difference between the metrics is that Temperature estimates the nestedness by the unexpected presence/absence of links in the empirical bipartite network with respect to the perfectly nested case for a fixed density (Remarkably, the fully nested structure implemented in the computation of the Temperature is a degenerate form where all the information can be obtained by the knowledge of the equilibrium shape, artificially setting the equilibrium diversification and ubiquity.). Instead, NODF estimates the nestedness by considering the degree of overlap that each row and column has with the others. Hence, an important difference between the two metrics is that the algorithm computing the Temperature requires the network to be re-ordered to achieve the most nested arrangement, while NODF is independent on the ordering. In the following we opt for the re-ordering given by the Fitness-Complexity ranks whenever we want to compute the Temperature, since the Fitness-Complexity algorithms has been shown to outperform other techniques^42^ in approximating the maximally nested arrangement.

Both measures of nestedness capture some feature of the dynamical evolution of the bipartite networks, and consequently of the innovation systems. For instance, by computing the overlap (co-occurrence) between countries, NODF can describe the ability of countries to follow the path of more developed economies, according to capability-based mechanisms. Indeed, NODF can be separated into the row and column components, allowing to disentangle the contribution of row-wise and column-wise co-occurrences to the nestedness of the system. On the contrary, Temperature evaluates the match and difference of the empirical network with a fully nested equilibrium of the environment, based on the stable state of a mutualistic system^37^. Therefore in the innovation systems, the Temperature is high when low performing countries are active in highly unexpected domains.

A problem encountered in the evaluation of the nestedness is that Temperature and NODF may depend on more basic topological properties of the networks that are not related to a particular visual pattern. For instance, the density of the network is the most important parameter in the estimation of the nestedness^40^, so that comparing networks with different densities is problematic. Another typical source of bias in the comparison of the nestedness is given by the degree distributions (the distribution of the diversification of countries and of the ubiquity of activities) since their evolution is not random and presents high temporal persistence. The standard way to correct this issue is to extract the statistical significance of the nestedness, scattering the empirical measures with respect to those obtained in suitable random models able to represent the selected biases^43,44^. For instance, the Erdos–Renyi (ER) null model^45^ draws a network ensemble constraining only the average density, while the Bipartite Configuration Model (BiCM)^39^ constrains also the average network degrees.

**Figure 3:**
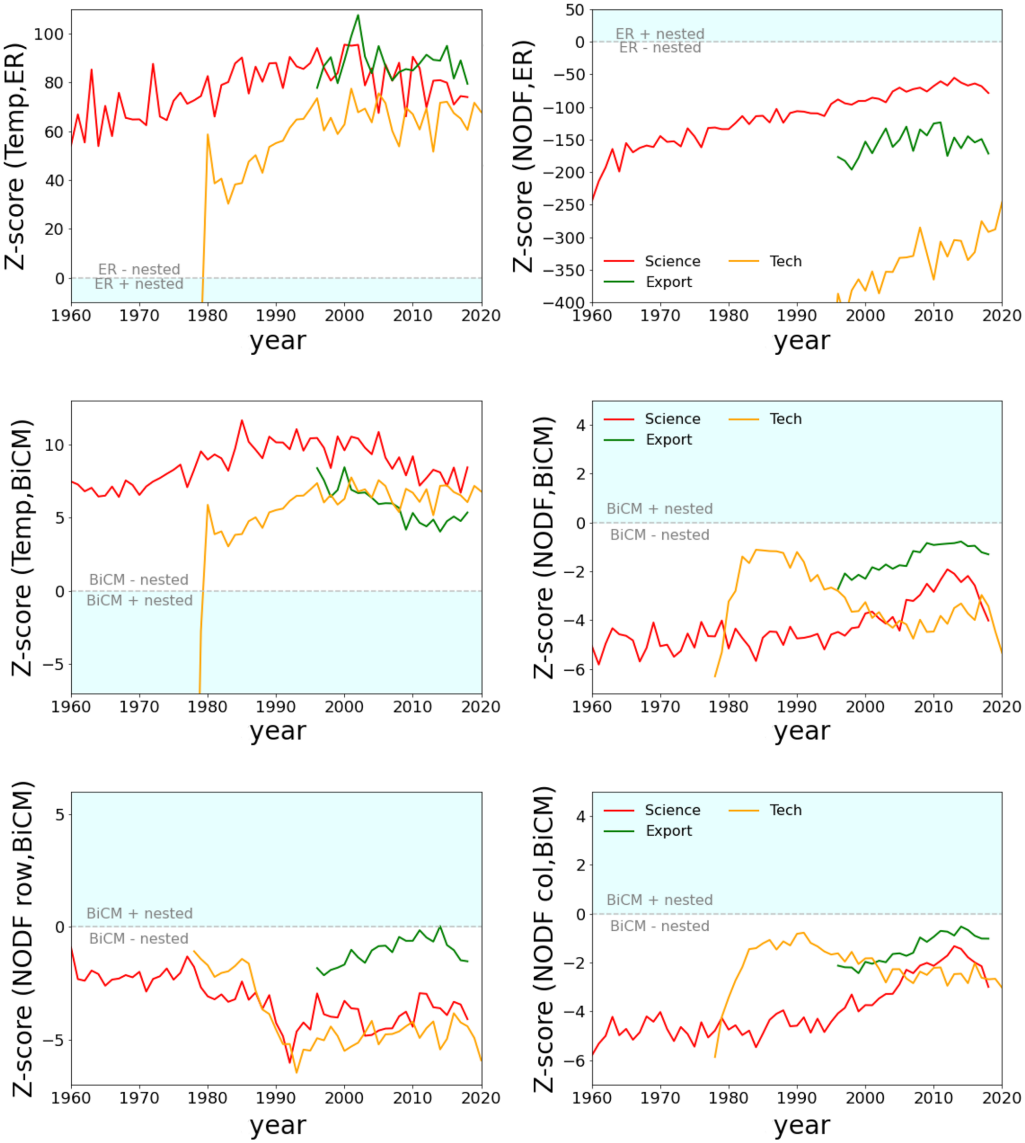
Temporal evolution of the Z-scores for the empirical measurements of nestedness (either Temperature or NODF) against a null network model (either ER or BiCM, which respectively constrain density and degree sequence). The different lines correspond to the various layers: Science (red), Technology (orange) and Trade (green). The light blue area marks the region of the plot where the null model is more nested than empirical data. The difference on the positions of the cyan regions derives from the inverted ranges of Temperature and NODF.

Irrespective of the increasing trend of both the density and the nestedness measures over time, discounting only the density does not provide much information in terms of evolution of the nestedness in the innovations systems. Indeed, the ER ensemble of matrices is much less nested than the empirical matrices, as shown in the top panels of Fig. 3.

According to the z-score values, which measures the distance of empirical values from ensemble averages in terms of ensemble standard deviations, in all layers empirical nestedness is much higher than in the random case. Instead, the null ensemble obtained by constraining the degrees of the nodes in the network with the BiCM, leads to a much lower significance of the features of the empirical network. Indeed, the value of the degrees is not random but has a persistent dynamics that affects the evolution of the nestedness, and this information must be accounted for in the null models. The information contained in the degrees is not usually taken into account in the biological literature on nestedness, where most of metrics were originally developed^46^ (see however^47,48^). For example, the perfect patterns for the Temperature are related only to a single parameter, the density. On the contrary, in the Economic literature, diversification (i.e. the node degree in the binary network) is the main feature against which the nestedness is studied^49^ (temporal series exploiting the dynamics of the systems can be obtained more easily in the economic literature, thus, it is possible that also in the ecological cases diversification could be important). However, the difference between the significance of Temperature and NODF is not strong and, in this analysis, the two nestedness measures correlate.

The nested pattern that emerges in innovation layers is usually related to a competitive dynamics driving capability accumulation. Instead, collaborative systems can create a more clustered network structures, with the growth of modules or communities^50^. In the biological realm, the modular organization of species interactions can increase the dynamical stability of the communities^51,52^ toward exogenous (external) perturbations. Instead, negative modularity might destabilize ecological systems. The combination of competitive and collaborative dynamics promotes the formation of local nested patterns or in-block nestedness, indicating the natural aggregation of components on the network.

**Figure 4:**
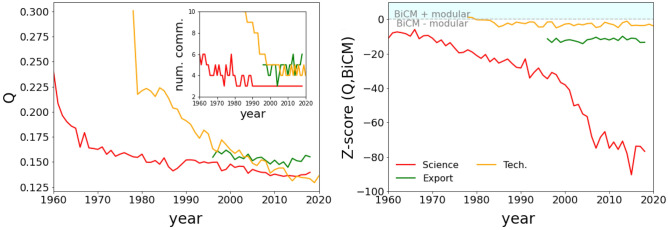
Left panel: the temporal evolution of the best modularity of the partition of countries within the different layers. The inset indicates the corresponding number of communities. Right panel: the temporal evolution of the z-scores of the modularity of the different layers. The line correspond to the Scientific (red line), the Technological (orange line) and the International Trade Export (green line). The light blue region on the diagram indicates the region where BiCM is more modular.

Focusing on the modular evolution of communities of countries having many internal connections (resulting from co-occurrences of activities) than connections with other communities, all the layers tend to level out at a comparable value of the optimal modularity, as seen in the left panel of Fig. 4. The decrease of the value of the best modularity is typically associated to a decrease in the quality of the modular structures^51,53^ due to a more uniform distribution of links within and across partitions. However, the effective number of modules, or communities, seems to converge to a number between 3 and 5, suggesting that the network is getting more and more clustered, as a result of a more globalized and collaborative world. Interestingly, the best partitioning of the scientific activities indicates neither a clear organization nor a block-nested structure. Indeed, only on the layer of countries a meaningful partition is found while the scientific fields are more homogeneously distributed, thus the emergent community structure is dependent on how different classifications separate the fields of science.

Following the same arguments discussed above regarding the nestedness, the modularity depends on the network’s features and the comparison of different networks is performed using the random model for the bias-removal. The right panel of Fig. 4 indicates that the modularity in science becomes more and more significant, thus the partitions are more and more reliable. On the contrary, both the international trade export and the technological layers are closer to a random structure, indicating that the modules are less meaningful and can be partially ascribed to the diversification and ubiquity.

## Discussion

In this section we discuss possible interpretations of the results.

Let us first focus on the evolution of the Scientific system. This highlights a constant growth of diversification: all countries are enriching their basket of active domains in science. Such growth persists from the end of the second world war. The countries with the most common capabilities become able to actively progress in more sophisticated research areas following the leading countries, while the leaders develop new fields of research increasing their diversification further. This picture is consistent with the standard narrative of the evolution of the whole innovation systems, from the Economic to the Technological layers, where the evolution of the patterns of national competitiveness is related to the evolution of capabilities brought by each single actor and country^54–56^. These models also have as a corollary that positive feed-backs in knowledge accumulation affects the further path of countries and firms: what you did in the past, defines what you know and therefore what you can do in the present^32,57–59^. The same conclusion can be drawn here because the most reliable patterns can be obtained by the null model built by fixing the diversification and ubiquity on the empirical values (see Fig. 3): past scientific capabilities of countries determines their accumulation. Instead, fixing only the global density, and therefore ignoring the individual dynamics of accumulation of capabilities, generates an ensemble where the average behavior is very far from the evolution of the empirical patterns.

Furthermore, the nestedness of the scientific network remains stable in the significant region considering both the Temperature and the NODF estimations, as seen in the central panels of Fig. 3. However, the binary matrix representation of the network indicates a new pattern hidden from the implementation of the citation counts metrics: two denser regions appear on the top right and bottom left sides with a hole in the top left corner. The appearance of two regions in the country layer suggests the emergence of a modular organization of economies that cannot be described by the sole dynamics of diversification, but needs to account a specialization mechanism. Thus, the region comprising the top rows is populated by the most diversified countries (the ones with the broadest set of capabilities) that expand in the most complex scientific fields while giving up some of their competitiveness in the less complex ones.

At the same time, the countries with narrower capability sets have more probability to acquire competitiveness in less sophisticated scientific fields, but continue lagging in the more complex sectors, as suggested by the fact that, according to the Temperature metric, the empirical network is significantly more nested than the random. A possible interpretation is that the evolution of the more advanced countries leads them to release resources from the less complex fields in favor of the most complex scientific fields; this could cause the appearance of the hole in the top left corner of the adjacency matrix, that is not contemplated by the standard narrative of diversification^54^. This hole is characterized by RCA values below the global average, which however remain close to the threshold. Therefore, in the scientific domain the driver of evolution is not simply the effort to achieve an increasing diversification of the basket of activities but, rather, a trade-off between diversification and specialization in order to better allocate the available resources. This behavior is probably the trademark of the scientific layer, where funding is usually channelled toward the most complex and highly-cited fields, instead of being broadly distributed throughout the spectrum of scientific activities. For instance, in the production of physical goods there is an economic advantage to produce also the less complex artifacts, while this feature is less important in the scientific realm. At the same time, the scientific evolution of the less performing countries is driven by emulation of the dynamics of leading countries with an increase of diversification, as indicated by the high significance of NODF. However, the significance of the Temperature suggests that, although the less performing countries are able to be active in some sophisticated fields, this is lower that the random expectation.

The Technological layer yields the visually best nestedness because the plot of the network’s adjacency matrix presents a clear triangular shape. The most diversifies countries lie at the top of the image with a roughly uniform basket of active sectors, ranging from the less to the most complex ones. The presence of a clear diversification among the countries suggest that the dynamics of the Technological environment follows the capability-based framework and for instance, NODF becomes more significant over time. Indeed, the NODF of rows (countries) follows that of the Scientific layer and the difference between the two relies on the different activities layers. Furthermore, the significance of the Temperature follows the same trend since the significant level of unexpectedness is comparable. Instead, the Technological environment is the less prone to create meaningful partitions, or modules, among the countries, and its dynamics is probably driven by the co-occurrence of knowledge and capabilities.

Finally, the Production environment revealed by export data displays a more conservative evolution, keeping roughly constant the network density and the effective number of active competitors in the system. Thus, the dynamics of the network is more related to an evolution of the nestedness pattern and not of the network size. Indeed, the nestedness follows the dynamics depicted by the other technological systems, highlighting the possible presence of common features of the dynamics of innovations. In general both for technologies and exports the expected specialization mechanism observed in the scientific field—while both theoretical expected and even empirically statistically significant when analysed carefully^60^—has a marginal and not significant role in the aggregate description of the system we observe in terms of nestedness. While scientific activities, constrained by a fixed budget, have to compete for resources with other fields *in the country*, this is not the case for export and technologies where a country with high capabilities can be hegemonic in many fields.

## Data Availability

The datasets and the code used and analysed during the current study available from the corresponding author on reasonable request.
